# Hybrid Oxford unicompartmental knee arthroplasty has lower residual cement extrusion than cemented arthroplasty in treating end-stage unicompartmental knee osteoarthritis

**DOI:** 10.1186/s12891-021-04720-9

**Published:** 2021-09-29

**Authors:** Guangzhong Yang, Xufeng Jiao, Qianli Li, Zheng Li, Shuai An, Mingli Feng, Guanghan Gao, Jiang Huang, Guanglei Cao

**Affiliations:** Xuanwu Hospital of Capital Medical University, 45 Changchun Street, Xicheng District, Beijing, 100032 China

**Keywords:** Unicompartmental knee arthroplasty, Hybrid, Cement extrusion, OUKA outcome

## Abstract

**Background:**

Hybrid Oxford unicompartmental knee arthroplasty (OUKA) consists of cementless femoral prostheses and cemented tibial prostheses. Although a hybrid OUKA has been used in clinical practice, the clinical outcome has not been reported. The purpose of this study was to compare the short-term clinical outcomes and rate of residual bone cement extrusion between hybrid and cemented prostheses and analyse the possible reasons for differences between outcomes.

**Methods:**

A total of 128 knees (118 patients) with end-stage osteoarthritis were included in this study, of which underwent consecutive operations using unicondylar Oxford phase 3 implants from July 2017 and September 2019 in our centre. Follow-up was performed at 6 weeks, 3 and 6 months, 1 year and every year after operation, and complications and changes in the Oxford knee score (OKS) were recorded. The OKS of the two groups was analysed by the generalized estimating equation approach. Prosthesis-based standard fluoroscopy was performed in a timely manner after each operation, and the rate of residual cement extrusion of the two groups was estimated using T-tests and a multivariate regression analysis.

**Results:**

Excluding the cases that lost follow-up, a total of 120 knees (65 in hybrid group and 55 in cemented group) were included in the analysis. There was no statistically significant difference in patient characteristics between the two groups (p > 0.05). The average follow-up time was 23.4 months (and ranged from 12 to 38 months). As of the last follow-up, there were no complications, such as dislocation, fracture, prosthesis loosening and subsidence, but one patient in the cemented group experienced symptoms caused by residual loose cement. Postoperative OKS in both groups improved significantly (p < 0.001). There was no significant difference in the OKS at any point during the follow-up or in the improvement of the OKS between the two groups (p > 0.05). Residual cement was mainly extruded behind the tibial prosthesis. The rate of hybrid periprosthetic residual cement extrusion was significantly lower in the hybrid group than in the cemented group, and the difference was statistically significant (OR = 3.38; p = 0.014).

**Conclusions:**

Hybrid OUKA is as effective as cemented OUKA in the short term after operation and can significantly reduce the residual cement extrusion rate around the tibial prosthesis.

## Background

Unicompartmental knee arthroplasty (UKA) has been used for the clinical treatment of unicompartmental osteoarthritis since the 1950s. Compared with total knee arthroplasty, UKA is characterized by a smaller skin incision and more musculoskeletal preservation, which maximally retains knee function and promotes rapid rehabilitation [1, 2]. The mortality and morbidity rates of UKA due to early complications are relatively low. Therefore, UKA has been receiving increasing attention and has been broadly applied. Promising clinical results have also been reported from studies on the application of UKA in some challenging settings [3, 4].

There are two types of UKA: mobile-bearing (Oxford UKA, OUKA) and fixed-bearing. OUKA was put into clinical practice in 1982, and its mobile bearing design can minimize wear and looseness [5]. Good long-term clinical results have been achieved in many high-volume OUKA centres [5–7]. Early designs of OUKA were fixed with cement. Registry data show that the revision rate of UKA is higher than that of total knee arthroplasty due to the learning curve, radiolucent lines and technical errors in cement application [8–11]. To reduce the revision rate, Oxford Designer launched cementless OUKA in 2004. This technique has now been in use for nearly 15 years and can produce excellent clinical results, including reducing the incidence of radiolucent lines, shortening the operative time, and minimizing the number of technical errors in cement application [12–15]. However, possibly because of differences in the morphology and anatomy of the tibia between Asian and Western populations, an increased fracture rate has been observed in the clinical application of biotype OUKA in Japan [16]. Thus, when this prosthesis was first launched in the Chinese market in 2017, a hybrid prosthesis – consisting of cementless femoral and cemented tibial components – was used. In our clinical application, we found that intraoperative cement removal was easier using hybrid OUKA than cemented OUKA, but there is a gap in the literature on the clinical results of hybrid OUKA. This prospective observational study was designed to compare the short-term clinical results of cemented and hybrid OUKA. We hypothesized that there was no significant difference in the short-term clinical results between the hybrid and cemented groups, and the hybrid group may have had less residual cement extrusion than the cemented group.

## Materials and methods

### Patients

In this prospective observational study, we compared the clinical results of 118 patients with end-stage unicompartmental osteoarthritis of the knee (corresponding to 128 knees) that underwent consecutive operations using unicondylar Oxford phase 3 implants from July 2017 and September 2019 in our centre. The subjects included 71 patients undergoing hybrid OUKA and 57 patients receiving cemented OUKA. The patients were allocated to a treatment group based on the type of intraoperative prosthesis used. All operative procedures were performed by the senior author (CGL), who is a high-volume arthroplasty surgeon with extensive experience in OUKA. All the patients provided informed consent, and the study was approved by the Ethics Committee of Xuanwu Hospital, Capital Medical University (approval number: 2017–091). All the procedures performed on human participants were carried out in accordance with the ethical standards of the institutional and/or national research committee and with the 1964 Helsinki declaration and its later amendments or comparable ethical standards.

Before the operation, anteroposterior (AP) and lateral X-rays of the knee; full-length weight-bearing, varus and valgus stress X-rays; and knee magnetic resonance imaging (MRI) scans were taken, and any patients with contraindications for OUKA were excluded. The inclusion criteria included medial unicompartmental knee osteoarthritis, as demonstrated by local “bone-on-bone” changes on X-ray; intact and functioning anterior cruciate ligament (ACL), posterior cruciate ligament, and medial collateral ligament; normal lateral compartment cartilage; varus deformity < 15°; and fixed flexion contracture deformity < 15°. The exclusion criteria included inflammatory joint disease, infectious disease, history of lower extremity fracture, and spontaneous osteonecrosis of the knee.

### Surgical method

Surgery was performed by an experienced surgeon using a hybrid or cemented Oxford unicompartmental prosthesis (two pegs, MP instrument, Biomet, Waterton Industrial Estate, Bridgend CF31 3XA, United Kingdom). Under direct vision during the operation, to ensure compliance with the indications, the ACL was required to retain intact function, and the lateral compartment cartilage lesion had to be diagnosed as grade 2 or less of the Outerbridge classification. Tibial and femoral preparation were performed according to Oxford standard operating procedures [17].

After femoral and tibial osteotomy, a spigot of an arbitrary size was inserted into the main hole and shaken properly. If there was no apparent shaking amplitude, a cementless femoral component was chosen, and a cemented femoral component was used otherwise. Before implanting the tibial component, a small quantity of cement was placed on the tibial surface and compacted into the bone with a 2-cm osteotome to increase cement penetration and create a thin layer of cement covering the surface. The insertion was completed by tapping the right-angled tibial impactor with a small hammer from posterior to anterior. Then, we implanted cement or the cementless femoral components (bone cement information: Biomet France SARL, Refobacin Bone Cement R).

Cement removal was performed thrice during prosthesis implantation, as described below.

The first excess cement removal was performed by using a nerve hook to remove cement spilled around the tibial component. Then, the femoral trial was inserted, and the cement was pressurized by inserting a feeler gauge to flex the knee to 45°. Then, the femoral trial component and feeler gauge were removed. The femur was lifted to obtain a clear view of the joint space, a 5-mm-wide osteotome was used to prop up the posterior capsule, and the nerve hook was used to carefully remove the excess cement from the back and medial sides of the component, corresponding to the second cement removal. After the femoral component was in place, a feeler gauge of an appropriate thickness was inserted, and the knee was maintained in flexion at 45° while the cement was setting. Once the cement was set, we removed the feeler gauge, carefully inspected the knee, and removed any residual cement, corresponding to the third cement removal. Note that femoral trials were used at this stage for hybrid prothesis. After the cement was set, we pulled out the trial and confirmed that all the residual cement had been removed. Pulsed lavage was employed to ensure that all visible excess cement had been meticulously removed.

### Postoperative management and rehabilitation

Local injection and an adductor canal block were used for analgesia. Prophylactic anticoagulant therapy and antibiotics were administered to prevent thrombosis and infection. To prevent thrombosis, patients wore compression bandages (elastic bandages) for 24 hours after operation and transitioned to wearing an antithrombotic elastic stocking for 6 weeks. All the patients began to perform lower-extremity muscle-contraction exercises on the day after the operation. Four hours after operation, patients was made to walk with a walker. A lower-limb pneumatic blood circulation pump was used during the night on the day of the operation. On the first day after the operation, knee joint exercises were performed with the assistance of a rehabilitation physician.

### Clinical and radiographic evaluation

Postoperative knee function was assessed at 6 weeks, 3 months, 6 months, 1 year and every year using the Oxford knee score (OKS).

**Fig. 1:**
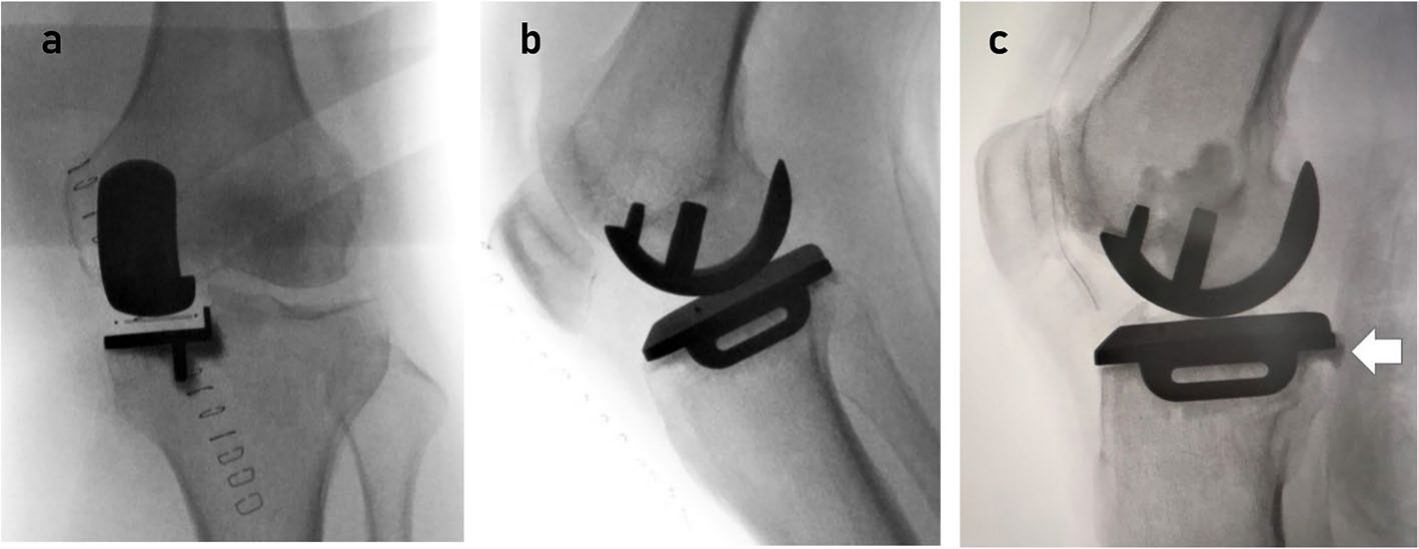
**A**. The surgeon manipulated X-ray beam and operative limb under fluoroscopic control until the X-ray beam was parallel to the side wall and base plate of the tibial component. **B**. The patient was positioned in a supine position with the knee flexed 30-40°.The fluoroscope was rotated through 90°so that the X-ray beam was centered on the femoral component. This meant that the X-ray was perpendicular to the side of the femoral component, so as to evaluate the residual cement around the prosthesis. **C**. One case of fixed excess cement of the posterior tibial component (arrow)

A C-arm X-ray machine was used to obtain AP and lateral views of the prosthesis-based standard unicompartmental fluoroscopy (Fig. 1) according to standard procedures that were subsequently analysed to evaluate the residual cement extrusion [17]. To reduce measurement error, the residual cement extrusion was separately evaluated by two independent investigators, and the pre- and postoperative X-rays were compared to prevent misdiagnosis caused by osteophytes and vascular calcification.

### Statistical analysis

SPSS (Version 21.0, IBM SPSS Statistics for Windows, Armonk, New York) was used for statistical analysis. The Pearson chi-square test was used to analyse categorical variables (such as sex and left-right side composition), and the t-test was used to compare the mean differences in continuous variables (such as the age, body mass index (BMI), operation time and duration of tourniquet application) between the two groups. The changes in the preoperative and postoperative OKS were analysed by the generalized estimating equation approach, and the residual cement extrusion was analysed by a logistic regression model. A p value < 0.05 was considered statistically significant.

## Results

A total of 128 OUKA cases were prospectively followed up in this study, and a total of 8 cases (6.3%) were lost to follow-up because the respective patients refused to participate in three consecutive phone investigations and did not keep additional clinic appointments within our system. Two patients (1 in the cemented group and 1 in the hybrid group) died of cardiovascular disease. Of those patients who were followed up in our outpatient department or by phone, 111 patients (23 males and 88 females, corresponding to a total of 120 knees) were analysed in this study. The mean age of the patients was 69.9 years (for a range of 51–89 years), and the mean BMI was 27.4 kg/m^2^ (for a range of 19.3–38.1 kg/m^2^). The average follow-up was 23.4 months (for a range of 12–38 months) postoperatively. The patients were classified based on the prosthesis type into a hybrid group (corresponding to 65 knees) and a cemented group (corresponding to 55 knees).

The results showed that there was no significant difference in the sex, age, BMI, proportion of left and right sides, or preoperative OKS between the two groups (p > 0.05). The average operation time was 73.9 minutes (for a range of 57–96 minutes) for the hybrid group and 74.4 minutes (for a range of 59–99 minutes) in the cemented group, with no significant statistical difference (p = 0.802). The average duration for the application of the tourniquet was 52.2 minutes (for a range of 41–67 minutes) for the hybrid group and 52.8 minutes (for a range of 38–68 minutes) for the cemented group, and there was no significant difference between the two groups (p = 0.58) (Table 1). A total of 12 patients in the hybrid group had distal deep venous thrombosis (below the popliteal vein), compared to 9 patients in the cemented group. There were no cases of proximal thrombosis or pulmonary embolism in either group.

**Table 1:** Mean Values for Demographic and Operative Variables, Along with the Statistical Comparison Between the Two Component Cohorts

Variable	Hybrid OUKA	Cemented OUKA	P. value
**Sex, n (%)**			
Male^a^	13 (50.0%)	13 (50.0%)	0.630
Female^a^	52 (55.3%)	42 (44.7%)	
Age, Mean (SD; range)	68.9(8.1; 51 to 89)	71.1(7.0; 54 to 89)	0.109
BMI, Mean (SD; range)	27.1(3.0; 19 to 32)	28.0(2.9; 22 to 38)	0.156
Operating time, Mean (SD; range)	73.9(9.7; 57 to 96)	74.4(9.6; 59 to 99)	0.802
Tourniquet time, Mean (SD; range)	52.2(5.7; 41 to 67)	52.8(5.7; 38 to 68)	0.580
**Side, n (%)**			
LEFT	32(47.7%)	29(52.7%)	0.703
RIGHT	33(52.3%)	26(47.3%)	
DVT	12	9	0.252

LEGEND: *BMI* body mass index, *DVT* deep vein thrombosis, *OUKA* Oxford unicompartmental knee arthroplasty

^a^ The number is based on cases of OUKA

**Fig. 2:**
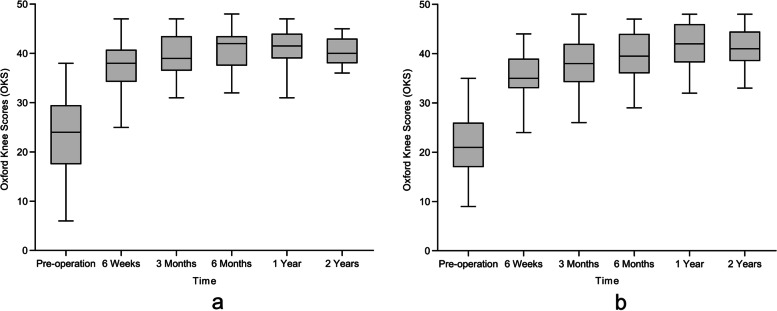
OKS score-time change trend of hybrid OUKA (**A**) and cemented OUKA (**B**)

The OKS at 6 weeks, 3 months, 6 months, 1 year and 2 years after the operation showed gradual improvement from the preoperative scores (Fig. 2). The OKS scores at different follow-up times were significantly different (P < 0.001) postoperatively. The OKS scores at 6 weeks, 3 months, 6 months, 12 months and 24 months after the operation improved to different degrees. There was no significant difference in the interaction between groups and time (P > 0.05), that is, there was no difference in the improvement of the OKS score between the two groups (Table 2).

**Table 2:** Analysis of Preoperative and Postoperative Oxford Knee Scores (OKS) Between Hybrid and Cemented OUKA Using Generalized Estimating Equation

	B	SE	95% CI	Wald Chi-Square	P
Constant	21.93	0.83	20.31 to 23.55	702.05	<0.001
Hybrid OUKA	1.49	1.25	-0.96 to 3.94	1.42	0.234
Cemented OUKA	0.00				
2 Years	19.19	1.06	17.12 to 21.27	328.32	<0.001
1 Year	19.77	1.28	17.28 to 22.27	240.69	<0.001
6 Months	17.95	1.15	15.69 to 20.20	243.11	<0.001
3 Months	15.57	1.09	13.43 to 17.72	202.94	<0.001
6 Weeks	13.37	1.17	11.08 to 15.66	131.44	<0.001
Pre-operation	0.00				
Hybrid OUKA *2 Years	-2.53	1.57	-5.60 to 0.55	2.60	0.107
Hybrid OUKA *1 Years	-1.95	1.60	-5.08 to 1.18	1.49	0.223
Hybrid OUKA *6 Months	-1.29	1.54	-4.32 to 1.74	0.70	0.404
Hybrid OUKA *3 Months	0.32	1.57	-2.76 to 3.40	0.04	0.837
Hybrid OUKA *6 Weeks	0.54	1.54	-2.48 to 3.57	0.12	0.726

LEGEND: *OUKA* Oxford unicompartmental knee arthroplasty

**Fig. 3:**
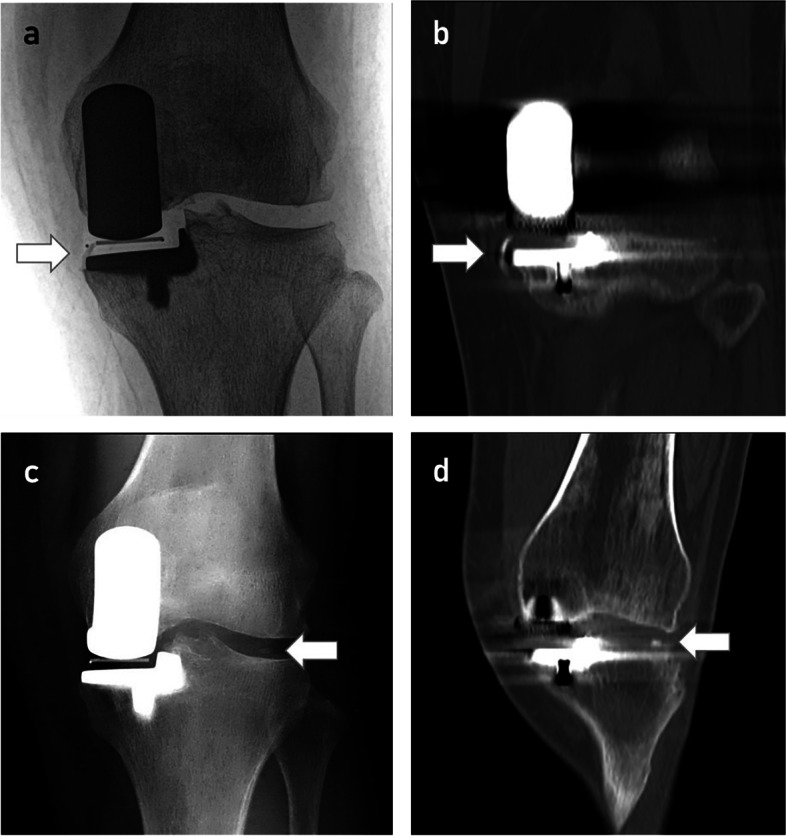
**A**.**B**. Postoperative standard unicompartmental fluoroscopy and CT showed residual cement (arrow) in the medial tibial prosthesis. **C**. **D**. When the patient developed symptoms 2 years after surgery, radiology revealed that the foreign body in the lateral compartment (arrow) was the same as the previous medial cement residue

**Fig. 4:**
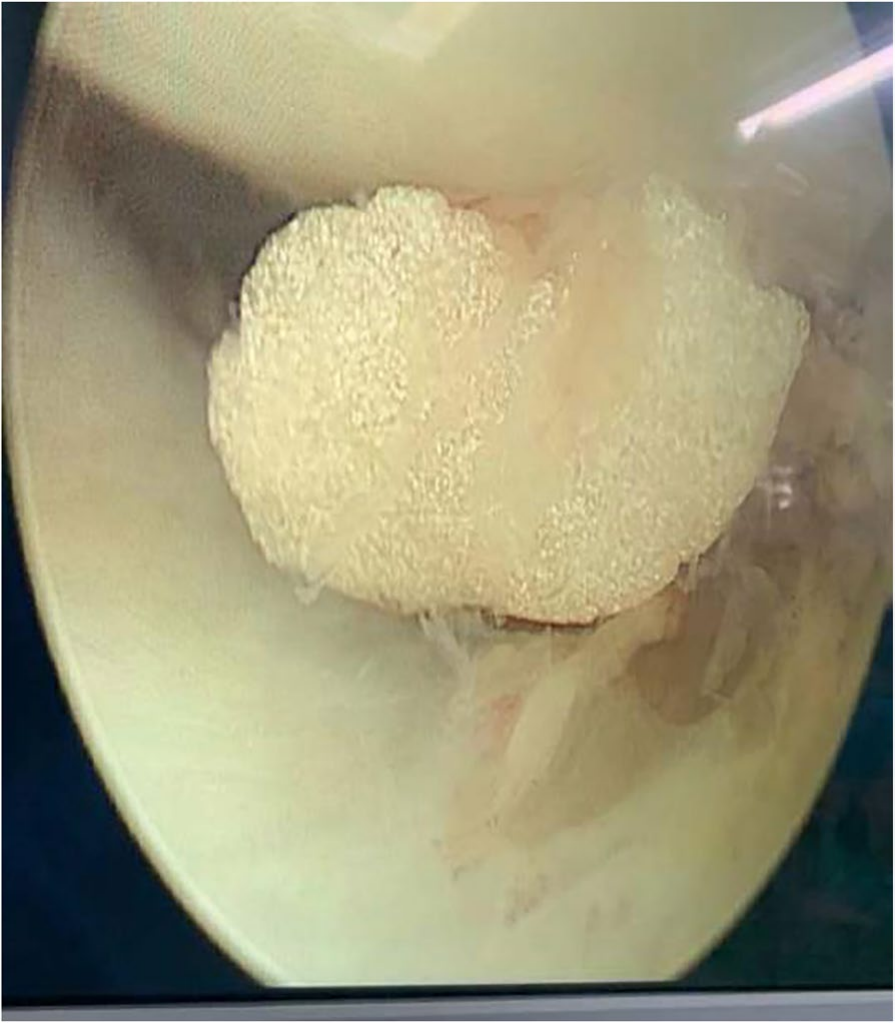
Arthroscopic removal of free cement body in the lateral compartment

Complications were caused by residual cement in one case: loose cement moved to the lateral knee compartment, which caused pain on the lateral side of the knee and reduced the range of motion at 2 years after the cemented OUKA was performed (Fig. 3). The free cement was removed by arthroscopy, upon which the pain on the lateral side immediately disappeared and the range of motion returned to normal (Fig. 4). Incision complications occurred in two cases in the hybrid group. One patient experienced incision leakage in the prepatellar bursa 10 days after operation. The other patient had skin incision dehiscence 3 months after operation due to a fall. Both patients were healed following debridement. There was also one case of an incision complication in the cemented group. The incision leaked 2 weeks after operation and healed following debridement. As of the last follow-up, there were no complications, such as dislocations, fractures, or prostheses loosening.

The presence of an radiopaque density extrusion shadow around the prosthesis and beyond the edge of the component or cortex in the X-rays was considered to indicate residual cement extrusion. The two observers were highly consistent in accurately identifying residual extrusion (interobserver reliability: 98.3%). In the hybrid group, there were 13 cases (20%) with residual cement extrusion, of which 10 had fixed residual cement behind the posterior tibial component, 2 had fixed residual cement near the medial tibial component, and 1 had a free cement fragment in the posterior joint space. In the cemented group, there were 21 cases (38.2%) with residual cement, of which 20 had fixed residual cement behind the posterior tibial component. In addition, 1 patient had a free cement fragment in the posterior joint space, 2 patients had fixed residual cement, and 1 patient had free cement near the medial tibial component. There was no fixed residual cement protruding above the surface of the tibial component. No residual cement was observed around the femoral component (Table 3).

**Table 3:** Situation of residual cement extrusion, along with the statistical comparison between the two component cohorts

	Hybrid OUKA	Cemented OUKA
Total	13(20.0%)	21(38.2%)
**The site of residual cement**		
Posterior tibial component	10(15.4%)	20(36.3%)
Medial tibial component	2(3.1%)	3(5.4%)
Posterior joint space	1(1.5%)	1(1.8%)
Femoral component	0	0

LEGEND: *OUKA* Oxford unicompartmental knee arthroplasty

The results of logistic regression model analyses showed that the rate of residual cement extrusion in the hybrid group was statistically lower than that in the cemented group (OR = 3.38; p = 0.014). The rate of residual cement extrusion behind the tibia was significantly lower in the hybrid group than in the cemented group (OR = 4.20; p = 0.005) (Table 4). Other factors, such as the BMI, age, surgical side and prosthesis size, did not affect the differences in the statistical results for the residual cement extrusion rate between the two groups.

**Table 4:** Logistic regression model analyses for cement residual of two groups using the possible influencing factors

Variable	OR	95% CI	P-Value
**Total Cement Residual**			
Prosthesis type	3.38	1.28 to 8.98	0.014^a^
BMI	0.99	0.94 to 1.05	0.811
Sex	1.27	0.30 to 5.44	0.745
Age	0.99	0.93 to 1.06	0.793
Prosthesis size	1.46	0.83 to 2.58	0.188
Side	0.55	0.21 to 1.44	0.220
Constant	0.20		0.652
**Cement Residual at Posterior tibial component**			
Prosthesis type	4.20	1.55 to 11.42	0.005^a^
BMI	0.10	0.95 to 1.05	0.895
Sex	1.47	0.33 to 6.58	0.616
Age	0.99	0.93 to 1.06	0.832
Prosthesis size	1.40	0.79 to 2.50	0.251
Side	0.69	0.26 to 1.86	0.464
Constant	0.08		0.498

LEGEND: ^a^ Statistically significant

## Discussion

The most important finding in this study is that the residual cement extrusion rate of the hybrid group was significantly lower than that of the cemented group, especially at the rear of the tibial component. In previous studies [18], the most frequently occurring site of cement residue after single arthroplasty was the posterior tibial prosthesis, which was consistent with the results of our study.

This observation may be mainly attributed to the blind spot that occurs during cement removal due to limited operation space, which may cause residual cement extrusion, especially behind the prosthesis. Although some degree of improvement can be achieved by adjusting the tools and procedure used, residual cement extrusion cannot be completely prevented. This situation is especially true of cemented prostheses, for which extrusion is more likely to occur when a package of cement is used to fix the tibia and femoral components simultaneously. Within this scenario, it is necessary to operate on the femoral side within 3–4 minutes after the start of mixing the cement to ensure adequate fixation strength [19]. Satisfying this time constraint requires the use of good cement technology on the tibial side. Residual cement extrusion is more likely to occur because of the difficulty of improving cement penetration and completely removing the bone cement. However, for hybrid prostheses, the aforementioned time limit to operate on the femur does not apply, leaving ample time to perform surgery on the tibial side, thereby ensuring full cement penetration and excess cement removal. The femoral trial can be removed after the cement on the tibial side has set, leaving a large operating space for the removal of cement hidden in the fold of the capsule on the tibia. Thus, compared to cemented OUKA, there is hybrid OUKA involves a larger operational space, an additional opportunity to perform cement removal and thus, a lower residual cement extrusion rate. The results of this study showed that the residual bone cement extrusion rate in the posterior tibia of the hybrid group was significantly lower than that of the cemented group, indicating that hybrid OUKA offers clear advantages in reducing the posterior tibial residual cement extrusion rate over cemented OUKA. The clinical application of hybrid prostheses could mitigate the problems caused by technical errors during cement application. Studies with larger sample sizes are needed to decrease deviations caused by statistical errors.

There have been few clinical reports on the residual bone cement extrusion rate after OUKA. The only existing study was performed by Hauptmann et al. [20] on 120 cases of cemented Oxford unicompartmental prostheses. Of the investigated cases, a total of 25 cases (21%) of residual excess cement were found, whereas 23 cases (19%) had free cement fragments. Hauptmann et al. considered that the limited operational space for OUKA promoted residual cement deposition and that the quantity of residual excess cement should not be underestimated. However, the X-ray film provided in the report was not a standard prosthesis-based anteroposterior or lateral view, which indicated that the residual cement deposition may have been underestimated in the study because cement may have been shielded by the component. In a cadaver study by Sheele et al. [21], the extrusion rate of residual free bone cement fragments after OUKA was found to be as high as 66.7%. Although there are differences between cadaver and clinical studies, the results of the cadaver study show that the extrusion rate of excess cement can be clinically underestimated. The extrusion rate of of excess cement in our study was higher than previously reported, at 36.3% in the cemented group and 20% in the hybrid group. This discrepancy may result from our use of standard anteroposterior and lateral view X-rays, which improved the detection rate and confirmed that the rate of residual bone cement extrusion could be underestimated. However, unlike Hauptmann’s study, there were only 3 cases of free bone cement fragments in our study, including 1 case in the hybrid group (2%) and 2 cases in the cemented group (4%). These case numbers are noticeably lower than those in Hauptmann’s study. We attribute the lower extrusion rate of free excess cement in our study compared to Hauptmann’s study to the cement removal strategy employed. That is, we performed the clearing operation thrice intraoperatively. The posterior capsule was propped with a narrow osteotome, and free cement fragments were removed with a nerve hook during the procedure. Therefore, although the total residual cement extrusion rate in our study was not low, the quantity of free bone cement was significantly reduced and there was no fixed bone cement residual on the surface of the prosthesis; the aforementioned two types of pathological residuals are the main causes of serious complications after OUKA [22–25].

For cemented prostheses, the time window for cement removal could be increased by fixing tibial and femoral prostheses separately but would increase the cost and the time for the bone cement to set, subsequently prolonging the duration of the application of the tourniquet. Consequently, the incidence of thrombosis and postoperative tourniquet-related thigh pain may increase [26, 27]. Our procedure of performing a deep vein ultrasound of the lower extremities 2 days after surgery may explain the increased incidence of DVT observed in this study. Ultrasonography can be effectively used to diagnose many asymptomatic DVTs, thereby increasing the DVT detection rate.

A study by Hiranaka [16] showed a relatively high incidence of tibial fracture using a cementless Oxford unicompartmental prosthesis in Japan, especially when small tibial components (e.g., A or AA sizes) were used. The results of research studies have shown that compared to European and American populations, the tibia of the Asian population is significantly different in shape and smaller [28, 29], such that the fault tolerance threshold for prostheses may be lower. The press-fit of the tibial keel is between 0.8 and 1.2 mm, which may place a high stress on a small tibia. The excessively short distance between the rear of the keel and a small posterior tibial component is more likely to interfere with the posterior cortex of the tibia when implanting a small component, which may increase the risk of fracture [30, 31]. In this study, an evaluation of the short-term postoperative knee function of hybrid OUKA prostheses showed that the OKS of the hybrid group on the postoperative trend chart was slightly higher than that of the cemented group during the early stages of the follow-up period (6 weeks and 3 months); however, the difference was not statistically significant. After 6 months, the results of the two groups were basically the same, and the OKS of the two groups continuously improved within 2 years after the operation. This result was similar to those of other studies on OUKA [32–34] and was consistent with our previous assumptions. In terms of complications, there were no periprosthetic tibial fractures during an average follow-up of 23.4 months in this study. Therefore, for the Chinese population, whose bone morphology is similar to that of Japan, it is reasonable to use a hybrid prosthesis until a cementless tibial component suitable for the Asian population is developed.

There are several limitations to our study. First, the study was not randomized. This limitation was caused by the prosthesis selection method. During the operation, the surgeon chose cement or cementless femoral prostheses according to the patient's bone quality and osteotomy, as well as an experimental installation of the prosthesis. This integrated process could not be standardized. Randomness did not affect the measurement of the main indicator, the rate of residual bone cement extrusion. Second, the C-arm X-ray machine was more likely to miss small residual cement fragments because of low resolution compared to that of postoperative X-rays and CT. However, postoperative standard AP and lateral radiographs are difficult to obtain, and CT image artefacts deteriorate screening accuracy. Moreover, the follow-up was short (46.7% of all OUKAs with at least two years of follow-up), and it was not possible to confirm the medium- and long-term impact of residual cement. However, it has previously been reported that clinical symptoms caused by residual bone cement mostly appear within 1 week to 1.5 years after operation [20, 23, 24]. Our follow-up covered the time period during which clinical symptoms are most likely to appear. Finally, as the sample size of this study was relatively small and a power analysis was not performed, large-scale randomized controlled trials with long-term follow-up are required to identify the long-term effects of the proposed treatment.

## Conclusion

Our study results showed that hybrid OUKA can produce short-term postoperative results as successful as those of cemented prostheses for treating end-stage medial unicompartmental osteoarthritis of the knee. The application of hybrid prostheses and a strategy of multiple removals of excess bone cement result in a reduction of bone cement residue around the tibial prosthesis.

## Data Availability

The datasets used and/or analyzed during the current study are available from the corresponding author on reasonable request.
